# OR3-002 – Blau Syndrome cohort study: ocular outcome

**DOI:** 10.1186/1546-0096-11-S1-A4

**Published:** 2013-11-08

**Authors:** CD Rose, R Cimaz, C Thomee, R Khubchandani, G Espada, R Russo, M Harjacek, B Bader-Meunier, P Brissaud, N Wulffraat, S Vastert, R Merino, A Naranjo-Hernandez, S Oliveira Knupp, F Mackenson, J Arostegui, J Anton Lopez, J Fernandez-Martin, C Wouters

**Affiliations:** 1 Nemours AI Dupont Hospital for Children, Wilmington, United States; 2 Università degli Studi di Firenze, Florence, Italy; 3 Centre Hospitalier de Luxembourg, Luxembourg; 4 Jaslok, Breach Candy and Saifee Hospitals, Mumbai, India; 5 Universidad Nacional de Buenos Aires, Buenos AIres, Argentina; 6 University of Zagreb, Zagreb, Croatia; 7 Hôpital Necker, Paris, France; 8 None, Neuilly/Seine, France; 9 UMC Utrecht, Utrecht, Netherlands; 10 Hospital La Paz, Madrid, Spain; 11 Hospital Dr. Negrin, Gran Canarias, Spain; 12 Universidade Federal do Rio de Janeiro, Rio de Janeiro, Brazil; 13 Universitäts Klinikum Heidelberg, Heidelberg, Germany; 14 Universidad de Barcelona, Barcelona, Spain; 15 Hospital do Meixoeiro., Vigo, Spain; 16 Katholieke Universiteit Leuven, Leuven, Belgium

## Introduction

BS is an autosomal dominant monogenic granulomatous disease due to gain of function mutations at or near the NACHT domain of NOD2. It is characterized by a triad of granulomatous polyarthritis, uveitis and rash. Retrospective work by our group showed a life time risk of ocular involvement of 60% with significant morbidity and poor visual outcome. Prospective studies on natural history of visual outcome are not available. In view of current lack of effective therapies, research on relevant pathways downstream NOD2 is essential and may lead to appropriate targeted drug development.

## Objectives

To study prospectively in detail the phenotype of ocular involvement and visual outcome in the context of a prospective cohort study on BS. Secondary goals: investigate possible biomarkers of disease activity and explore relevant pathways and candidates for therapeutic targeting.

## Methods

Participating centers of an ongoing international registry were invited to enroll patients with NOD2 mutation. IRB approval was obtained. This 3 year prospective study consists of one baseline and 3 yearly assessments with a standardized clinical evaluation, functional assessment, visual analogue scales, a comprehensive ophthalmologic assessment and blood sampling for fundamental in vitro research. Coded data are kept in a secured database at the coordinating center.

## Results

We are reporting baseline ophthalmologic evaluation of the first 23 patients, virtually a cross section of ocular status along disease course. Ages were 0-54 years. 50% 0-15. More than half had substitutions R334W or R334Q. 19/23 have ocular involvement. Onset of eye disease was 67 months (6-264), 30 months after the onset of arthritis. Uveitis never preceded joint disease, was bilateral in 90% and "pan" in 15/19. Despite intense therapy there was evidence of active disease (+ cells and/or flare for anterior segment or macular edema) at the time of evaluation in 15/19 patients, (anterior in 8, posterior in 4 and global in 3).For severity assessment in bilateral disease we used the worse eye. Disease was mild if no local complications (except cataracts), moderate when complicated and severe if there was visual loss (WHO). Accordingly 2 were mild, 6 moderate and 11 severe. Corrected visual acuity (10=100%) was poor, with an average of 7.1 for the right eye and 6.7 for the left.

## Conclusion

Eye involvement in Blau disease is common, severe, requires intense therapy and lends significant impact on morbidity. This first prospective cohort multicenter study of Blau syndrome shows that development of effective therapies with bioactivity in ocular tissue is critical.

## Disclosure of interest

None declared

